# Single-molecule long-read sequencing reveals the potential impact of posttranscriptional regulation on gene dosage effects on the avian Z chromosome

**DOI:** 10.1186/s12864-022-08360-8

**Published:** 2022-02-11

**Authors:** Jianmei Wang, Yang Xi, Shengchao Ma, Jingjing Qi, Junpeng Li, Rongping Zhang, Chunchun Han, Liang Li, Jiwen Wang, Hehe Liu

**Affiliations:** Farm Animal Genetic Resources Exploration and Innovation Key Laboratory of Sichuan Province, College of Animal Science and Technology, Sichuan Agricultural University, Chengdu, 613000 China

**Keywords:** Nanopore, Posttranscriptional regulation, Sex chromosomes, Dose effects, Duck

## Abstract

**Background:**

Mammalian sex chromosomes provide dosage compensation, but avian lack a global mechanism of dose compensation. Herein, we employed nanopore sequencing to investigate the genetic basis of gene expression and gene dosage effects in avian Z chromosomes at the posttranscriptional level.

**Results:**

In this study, the gonad and head skin of female and male duck samples (*n* = 4) were collected at 16 weeks of age for Oxford nanopore sequencing. Our results revealed a dosage effect and local regulation of duck Z chromosome gene expression. Additionally, AS and APA achieve tissue-specific gene expression, and male-biased lncRNA regulates its Z-linked target genes, with a positive regulatory role for gene dosage effects on the duck Z chromosome. In addition, GO enrichment and KEGG pathway analysis showed that the dosage effects of Z-linked genes were mainly associated with the cellular response to hormone stimulus, melanin biosynthetic, metabolic pathways, and melanogenesis, resulting in sex differences.

**Conclusions:**

Our data suggested that post transcriptional regulation (AS, APA and lncRNA) has a potential impact on the gene expression effects of avian Z chromosomes. Our study provides a new view of gene regulation underlying the dose effects in avian Z chromosomes at the RNA post transcriptional level.

**Supplementary Information:**

The online version contains supplementary material available at 10.1186/s12864-022-08360-8.

## Introduction

Sex chromosomes often lead to the difference of sex-linked gene dose between the sexes; if this difference is not compensated for, it will have a massive impact on the organismal evolution [1–3]. For the female homogametic species (females XX, males XY), sex chromosome dosage compensation is regarded as a means to balance the expression levels of sex-linked genes in the two sexes. For instance, in *Drosophila*, the transcription of the genes located in the single X chromosome in males is upregulated [4, 5]. In mammals, the genes of the X chromosome are largely inactivated in females [6, 7]. In *C. elegans*, dosage compensation is achieved by the dosage compensation complex (DCC), which binds to both X chromosomes to downregulate X-linked gene expression in hermaphrodites [8, 9].

However, regarding male homogametic species (males ZZ, females ZW), such as birds, recent studies reported that ZZ/ZW system species lacked global sex chromosome dosage compensation [10], and higher transcription levels of Z-linked genes were observed in males than females [11–13]. Additionally, studies have also primarily focused on time- and tissue-specific expression changes. For example, the sex chromosome dose-effect of genes exists, and tissue-specific, sex-biased expression tends to be high in the gonads but lower in other tissues [14]. Among tissue time-point combinations, the embryonic brain had the smallest gene dosage effects, and adult gonadal tissue had the largest degree of male bias [15, 16]. These results indicated that the gene dosage effects of avian sex chromosomes are generally maintained in highly differentiated and adult tissues. Furthermore, studies also suggested that some genes were individually dosage compensated, and some were local or temporal dosage compensations on the avian Z chromosome [17, 18].

At present, the transcriptional regulation of gene expression on avian sex chromosomes has been extensively studied. Nonetheless, the posttranscriptional regulation of gene expression is very complex, including the regulatory mechanisms at the levels of premessenger RNA (mRNA) processing (capping, splicing, and polyadenylation), mRNA stability, and mRNA translation [19, 20]. To date, more than 170 post-transcriptional of RNA modifications have been identified [21], including messenger RNAs (mRNAs), transfer RNAs (tRNAs), ribosomal RNAs (rRNAs) and long noncoding RNAs (lncRNAs) [22–24]. Despite all this, the potential impact of posttranscriptional regulation modifications, such as alternative splicing (AS), alternative polyadenylation (APA) events and long noncoding RNAs (lncRNAs) on the gene dosage effects of the avian Z chromosome remains largely unknown [10, 25–27]. However, reads derived from current short-read RNA sequencing (RNA-Seq) technologies are usually short and deprived of information on modification, influencing their potential in defining transcriptome complexity [28, 29]. In contrast, there are many advantages in the Oxford Nanopore Technologies (ONT) full-length transcriptome, which allows a comprehensive analysis of transcriptomes in identifying full-length splice isoforms and several other posttranscriptional events, including long noncoding RNAs (lncRNAs), alterable splicing (AS), and alternative polyadenylation (APA).

Therefore, the duck was taken as the primary research object in this work, using Nanopore sequence technology to explore the potential impact of posttranscriptional regulation on gene dosage effects on the avian sex chromosome. These findings provide new knowledge of the gene regulation underlying the dose effect of avian sex chromosomes at the RNA posttranscriptional level.

## Results

### Overview for the ONT data

We used ONT to compare the transcriptome differences of gonad and head skin tissues between males and females. The head skin are highly sexually differentiated tissues. The head of male duck is green, while that of female duck is dull. With four biological replicates, the transcriptome sequencing of 16 duck samples generated a total of 3.92 Gb clean reads, with a base calling accuracy > 90.00% and coverage > 85.00% per library, and with values ranging from 79.30 to 84.95% full-length reads were obtained (Additional file 1: Table S1). In addition, rarefaction analysis showed that sequencing depth, achieved using PromethION flow cells, was nearly saturated with the number of transcriptomes discovered within those size ranges (Additional file 4: Fig. S1). Subsequently, the clean reads were subsequently mapped to the duck reference genome (GCF_015476345.1, 2020).

**Fig. 1:**
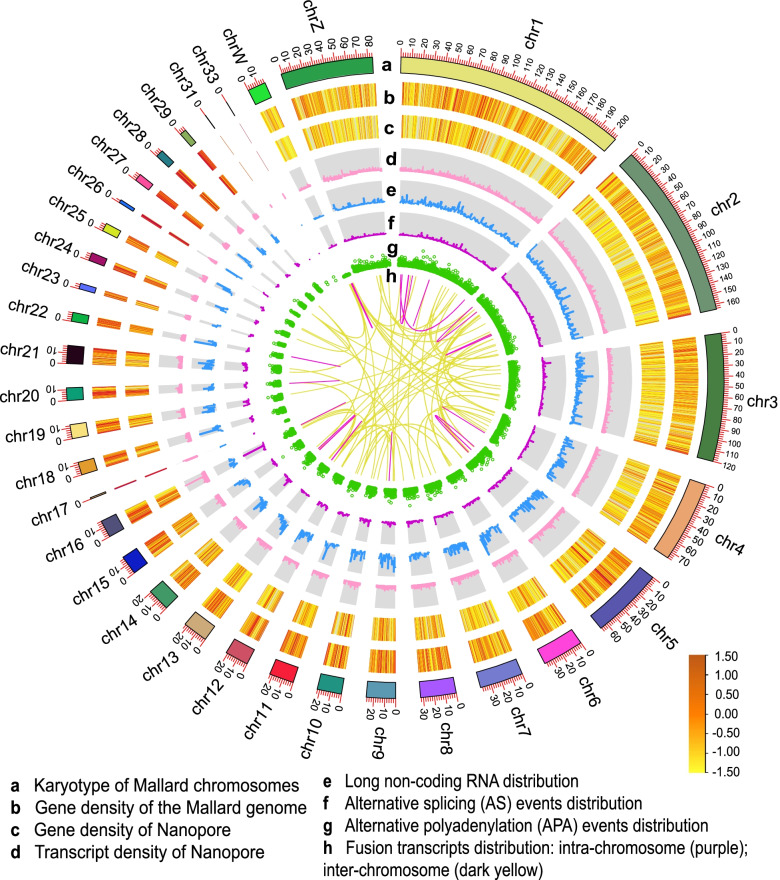
Circos visualization of the duck genome and Nanopore results. **a** Karyotype of the mallard chromosome; **b** Gene density of the mallard genome; **c** Gene density of nanopores; **d** Transcript density of nanopores; **e** Long noncoding RNA (lncRNA) distribution; **f** Alternative splicing (AS) event distribution; **g** Alternative polyadenylation (APA) event distribution; and **h** Fusion transcript distribution: intrachromosome (purple); interchromosome (dark yellow). The distribution was calculated in a 1-Mb sliding window at 10 kb intervals

The duck genome and ONT data information are provided as follows (Fig. 1): 23,807 gene loci and 83,483 transcripts were found (Fig. 1c and d). In addition, 6687 lncRNAs were marked with four computational approaches (Fig. 1e). In addition, a total of 16,836 AS events were detected, and they were further subdivided into five main types (Fig. 1f). A total of 15,862 APA events were identified using the TAPIS pipeline (v1.2.1) (Fig. 1g). A total of 443 fusion transcripts were identified (Fig. 1h). Fusion events were more likely to occur interchromosomally than intrachromosomally, and they tended to occur near chromosome termini.

### Relative sex-biased expression levels of genes on the Z chromosome

**Fig. 2:**
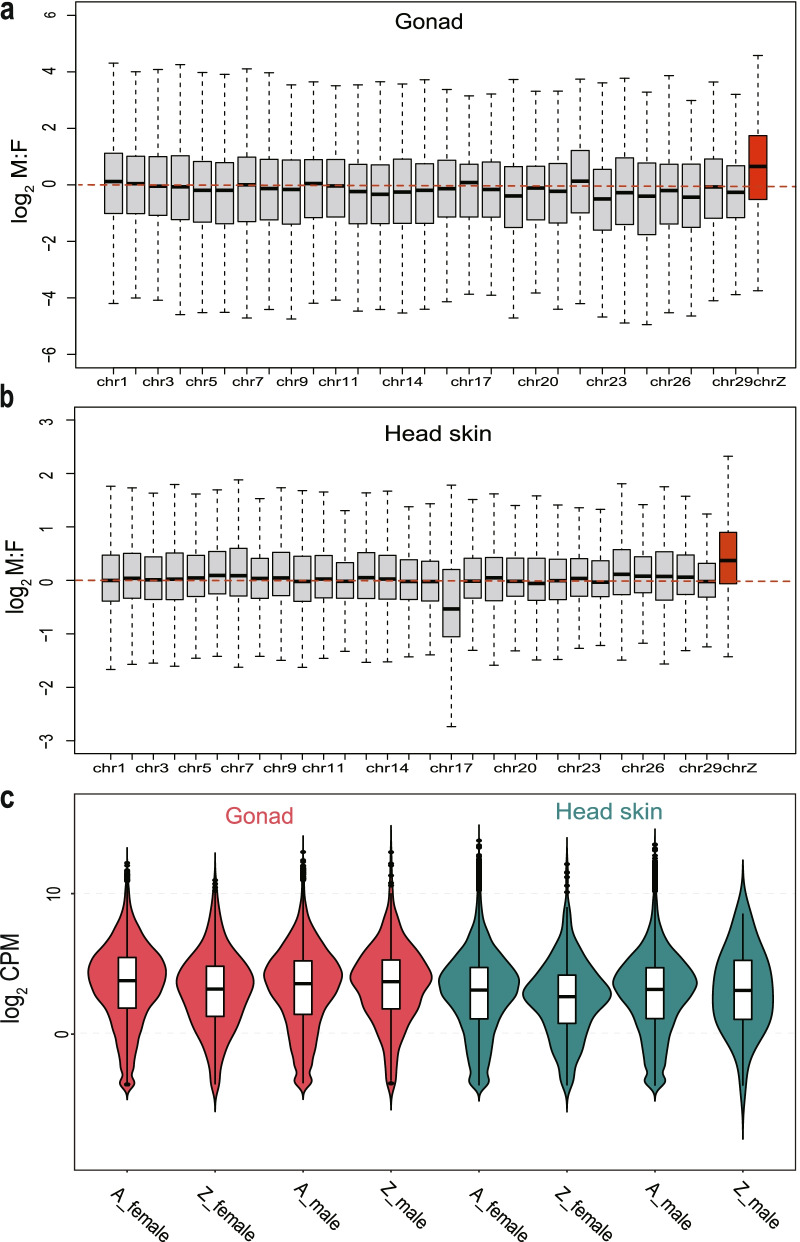
Comparison of the expression levels of autosomal-linked and Z chromosome-linked genes (CPM > 0). The M:F (male:female) ratio distributions on the Z chromosome and the autosomes for the gonad (**a**) and head skin (**b**). **c** Violin plots of the Z chromosome and autosome gene expression for each tissue in males (ZZ: AA) and females (Z: AA). Read counts were converted to counts per million (CPM) values to measure the expression of genes. Values are represented on a log_2_-transformed CPM scale (outliers of |log_2_ FC| > 5). For simplicity, chromosomes W, 31, and 33 are not included. The red lines indicate equal expression levels between females and males on the chromosome. **** *P* < 0.01, ns *P* > 0.05

The M:F ratios (sex-biased expression of autosomal and Z-linked genes) and Z(Z):AA ratios (autosomal relative expression of Z-linked genes) were used for further analysis. To verify the existence of incomplete dosage compensation in ducks, we compared the log_2_ M:F (male:female) ratios of genes expressed on autosomes plotted together with the Z chromosome. In gonad and head skin tissues, the log_2_ M:F (male:female) ratio of autosomes is close to zero. In contrast, the log_2_ M:F (male:female) ratio of the Z-linked genes showed a significant male bias, with a 1.73-fold higher expression in the gonads of males than females and a 1.78-fold higher expression in the head skin tissues of males than females (Fig. 2a, b). This finding suggested that the Z-linked genes were not well balanced and compensated between males and females, supporting a dosage effect exiting between the two sexes.

Furthermore, the Z(Z): AA ratio analysis showed significantly lower expression of Z-linked genes than autosomes in the gonads of females (*P* = 1.7e^− 07^, Wilcoxon rank-sum test) and head skin of females (*P* = 6.8e^− 05^, Wilcoxon rank-sum test). In contrast, our results confirmed no significant difference in expression level between Z-linked genes and autosomal genes in the gonads of males (*P* = 0.1, Wilcoxon rank-sum test) and head skin of males (*P* = 0.73, Wilcoxon rank-sum test), respectively (Fig. 2c).

### Regional differences in sex-biased gene expression on the Z chromosome

**Fig. 3:**
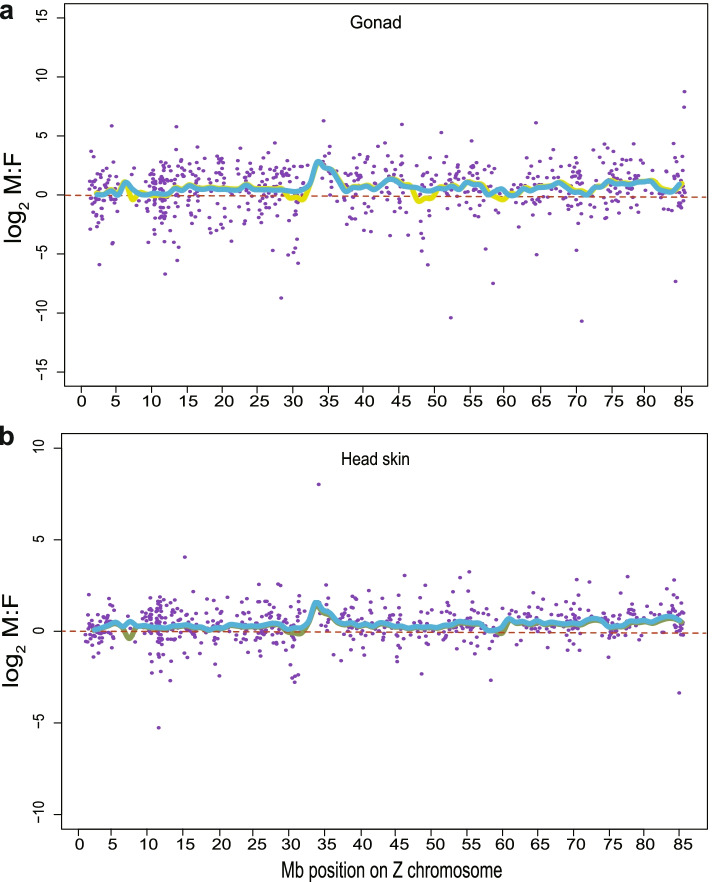
Fold-change map of the duck Z chromosome for gonads (**a**) and head skin (**b**). log_2_ M:F (male:female) ratios were plotted for each gene. Green lines represent the average fold-change for overlapping windows. Blue lines represent the moving average for amplitude. The amplitude metric was the absolute value of the log_2_ M:F (male:female) expression rate. The higher the amplitude is, the greater the degree of sex bias

A total of 576 Z-linked genes were identified in our data. To verify whether any genes were locally specific to dosage compensation on the duck Z chromosome, we used a 3 Mb window and a 1 Mb shift to calculate the moving average fold-change and amplitude for each tissue (Fig. 3**)**. As a result, the 31–36 Mb region on the Z chromosome was identified as the region with particular sex-biased gene expression for both gonad (average log_2_ M: F (male: female) = 2.05) and head skin tissues (average log_2_ M: F (male: female) = 1.45) (Fig. 3a, b), indicating that the sex-biased expression of genes in this region was the best among the whole Z chromosome. Thus, the above results demonstrated that the regulation of sex-biased gene expression was regional on the duck Z chromosome. In addition, GO and KEGG pathway enrichment analyses showed that male-biased gene expression was mainly enriched in cellular components and metabolic pathways in both tissues (Additional file 5: Fig. S2a, b).

### Discovery of sex-biased transcripts and AS events on the Z chromosome

Some genes might splice as multiple transcripts resulting from alternative splicing. The expression level of the transcripts showed that in both tissues, the M:F (male:female) ratio of autosomes is close to zero on the log_2_ scale. However, the log_2_ M:F (male:female) ratio of the Z-linked transcripts showed 0.64-fold higher expression in males than females for gonad tissues, with 0.74-fold higher expression in males than in females for head skin tissues (Additional file 6: Fig. S3a, b). Moreover, the Z(Z): AA ratios indicated significantly low expression of Z-linked transcripts compared to autosomes in the gonads of females (*P* = 2.22e^− 16^, Wilcoxon rank-sum test) and head skin of females (*P* = 1.1e-^10^, Wilcoxon rank-sum test). In contrast, the expression of Z-linked genes was not significant compared to autosomes in the gonads of males (*P* = 0.09, Wilcoxon rank-sum test) and head skin of males (*P* = 0.65, Wilcoxon rank-sum test) (Additional file 6: Fig. S3c).

Of the 576 Z-linked genes, approximately 283 (49%) genes had more than two different transcripts (Additional file 7: Fig. S4a). Venn diagram showing the tissue specificity of Z-linked transcripts. A total of 38 and 34 unique transcripts were identified in the gonads of males and females, respectively (Additional file 7: Fig. S4b). A total of 31 and 51 unique transcripts were identified in female and male head skin, respectively (Additional file 7: Fig. S4c). GO analysis showed that unique transcripts are enriched for particular molecular functions that vary with tissues. In the gonads of male and female, unique transcripts (72) were enriched for the cellular response to hormone stimulus. In contrast, unique transcripts (82) were enriched in catalytic activity in head skin of male and female. KEGG pathway enrichment analysis showed that tissue-specific transcripts were enriched in metabolic pathways in both tissues (Additional file 7: Fig. S4d).

**Fig. 4:**
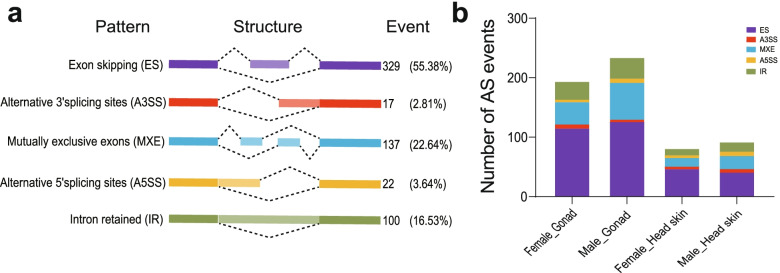
Comparison of alternative splicing modes on the duck Z chromosome. **a** Visualization of five alternative splicing modes. **b** Distribution of different types of alternative splicing event sampling among tissues

Most importantly, AS is a regulatory mechanism for the diversity of transcripts. We ascertained the five principal modes of alternative splicing. Of these, exon skipping accounted for 55.38% of alternative transcripts that were the most enriched type using AStalavista [30] (Fig. 4a). Then, we also found that the distribution of these five types of AS events in each tissue was different (Fig. 4b), with AS events higher in males than females for transcripts. Of these, 195 and 235 alternative splicing events were identified in female and male gonadal, respectively, and 80 as were overlapped events. Eighty-two and ninety-three alternative splicing events were identified in female and male head skin, respectively, and 51 as events overlapped.

**Fig. 5:**
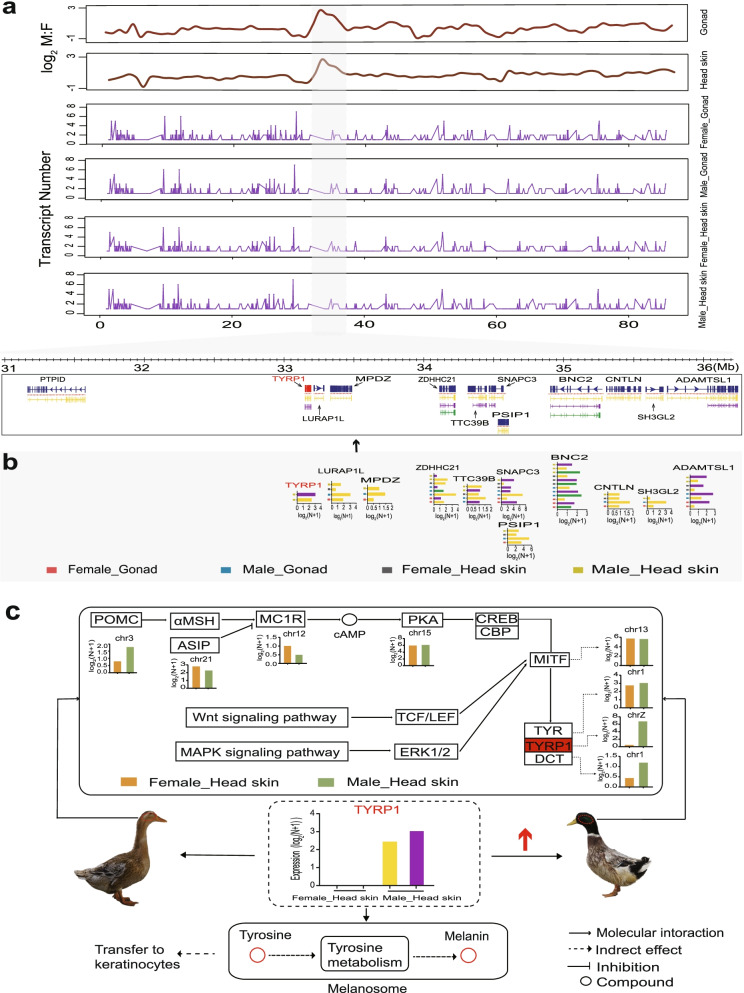
Comparison of different transcripts at the duck Z chromosome position. **a** Different transcripts of the duck for four tissues at the Z chromosome position. **b** Transcript of male sex-biased expression in different tissues for the 31–36 Mb region. Transcript expression test for log_2_ (CPM + 1) value. **c** Gene pathway associated with melanin synthesis. The duck images were taken with an Olympus DP70 digital camera (Center Valley, PA), the digital camera was set to a manual exposure manner, and each photo was based on identical exposure conditions, including exposure time and aperture

Moreover, there were differences in the number of transcripts in the 31–36 Mb regions (Fig. 5a). In this region, the expression of transcripts per gene existed for sex-biased expression among tissues (Fig. 5b). GO and KEGG enrichment analyses suggested that the main functions of sex-biased transcripts were involved in the melanin biosynthetic process. The most significant pathways were enriched in tyrosine metabolism and melanogenesis (Additional file 8: Fig. S5). Notably, the TYRP1 gene was a critical downstream gene of three melanin synthesis pathways (MC1R-cAMP, Wnt, and MAPK) (Fig. 5c). Additionally, the results of gene expression in this pathway showed that only the TYRP1 gene was more differentially expressed in males than in females (102 vs. 0.4). Indeed, our study also identified that only two transcripts of the TYRP1 gene were expressed in male head skin. Thus, the above result suggested that TYRP1 could be associated with pigment deposition in male ducks.

### Sex-biased long noncoding RNA (lncRNA) identification on the Z chromosome

**Fig. 6:**
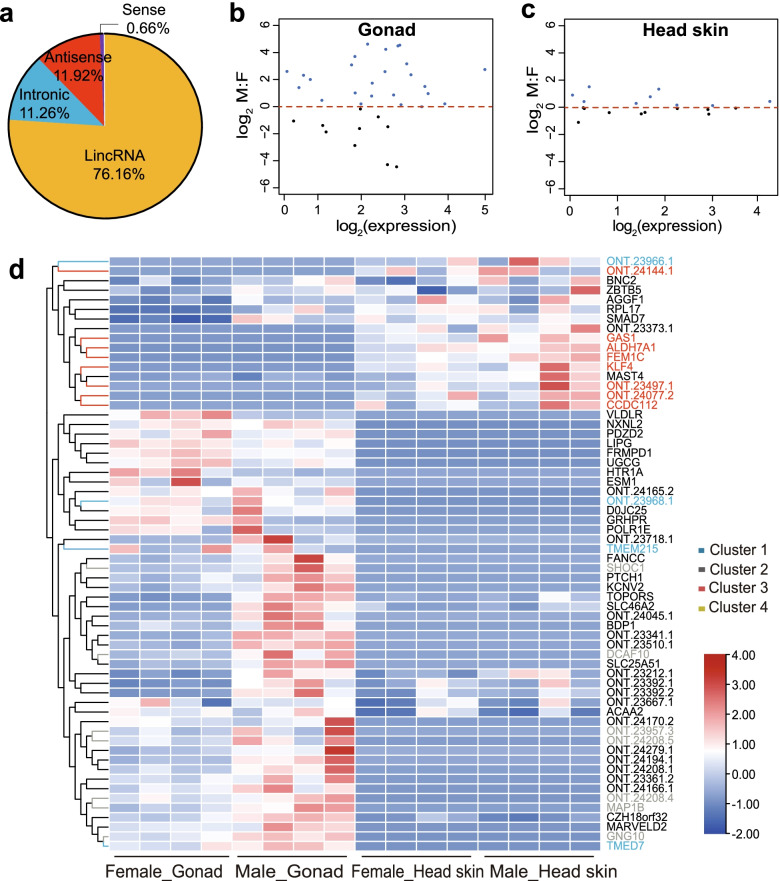
Characteristics of identified lncRNAs on the duck Z chromosome. **a** Proportions of four types of lncRNAs classified based on biogenesis. Male-biased lncRNA expression (log_2_ M: F > 0) in gonads (**b**) and head skin (**c**). **d** Heatmap of lncRNAs and their Z-linked target gene expression per sample

In addition to protein-coding RNAs, noncoding RNAs also constitute a significant component of the transcriptome. A total of 151 lncRNAs on the Z chromosome were divided into four categories: 76.16% were generated from intergenic regions (lincRNAs), 11.92% from antisense strands (antisense lncRNAs), 0.66% from sense strands (sense lncRNAs), and 11.26% from intronic regions (intronic lncRNAs) (Fig. 6a), based on their positions in the reference genome (GCF_015476345.1, 2020). The log_2_ M:F (male:female) ratio of the Z-linked lncRNA showed 19 lncRNAs with male-biased expression in the gonad and 9 lncRNAs with male-biased expression in the head skin (Fig. 6b, c). Then, based on the position (< 100 kb upstream and downstream) and base complementarity of lncRNAs, a total of 44 and 23 Z-linked target genes of 19 lncRNAs in the gonad and 9 lncRNAs in the head skin were predicted, respectively (Additional file 2: Table S2). Expression profiling confirmed that male-biased lncRNAs and their Z-linked target genes exhibited tissue-specific expression (Fig. 6d). Gene expression clustering analysis showed that Z-linked target genes were positively regulated through male-biased lncRNAs in each tissue (Additional file 9: Fig. S6a). Furthermore, GO and KEGG enrichment analyses suggested that the target genes of male-biased lncRNAs were enriched in the cellular component of both tissues. The most significant pathways were enriched in the metabolic pathway (Additional file 9: Fig. S6b). Finally, we also found that the distribution of log_2_ M: F (male: female) lncRNA expression on each chromosome was different in gonad (Additional file 10: Fig. S7a) and head skin (Additional file 10: Fig. S7b).

### Alternative polyadenylation (APA) events on the Z chromosome

**Fig. 7:**
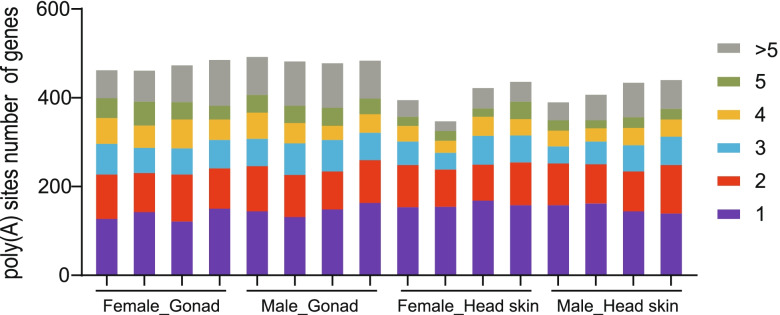
Distribution of the number of poly (A) sites per gene. Colored bars represent the number of poly(A) sites per gene

Alternative polyadenylation (APA) is also a mechanism of diverse gene expression similar to alternative splicing. We used the TAPIS pipeline to detect APA events per sample. The qualified gene loci for APA must be supported by at least two aligned FLNC reads. Of the 576 Z-linked genes with evidence of a poly(A) site. The Z-linked genes of 381 (72.71%), 368 (71.18%), 269 (59.38%), and 307 (66.16%) were observed to have two or more detected poly(A) sites in the testis, ovary, head skin of females, and head skin of males, respectively (Fig. 7). This result suggested that alternative polyadenylation (poly-A) exhibited differences in each sample. Whereas, there were no statistically significant differences between males and females (*P* > 0.05).

## Discussion

Studies of gene expression on the Z chromosome in avians have shown that they lack a global mechanism of dosage compensation to equalize gene expression between sexes on the Z chromosome. Sex-biased gene expression is much more prevalent on the avian Z chromosome than on autosomes [11–13], and regulation of localized dosage compensation is likely to take place by a gene-by-gene process that varies between tissues and time points [14–17]. Previous studies mainly focused on chickens showed that the gene dosage effects of the avian Z chromosome could be detected in the gonad and other tissues, such as the brain, muscle, heart, and liver [14–17]. Moreover, our study is the first to focus on highly differentiated adult duck gonad and head skin tissues to obtain differences in the gene expression of sex chromosomes between males and females. Our results similarly indicated a dosage effect and local regulation of ducks’ Z chromosome gene expression, which validated findings from previous research.

However, gene expression is modulated at many levels, including transcription, posttranscriptional RNA modifications (RNA splicing and export), RNA stability, and finally, mRNA translation [31]. RNA modifications play essential roles in the regulation of gene expression [32]. Until now, over 170 kinds of RNA modifications have been identified, which have allowed expanding the RNA code [33]. We used nanopore sequencing to confirmed the important modes of posttranscriptional regulation, such as alternative splicing (AS), alternative polyadenylation (APA) and long noncoding RNAs (lncRNA), all of which play an important role in regulating gene expression and dosage effects on duck Z chromosomes. Mittleman et al. demonstrated that the main mechanisms contributing to mRNA transcript diversity are alternative splicing (AS) and alternative polyadenylation (APA) [34]. AS allows a single gene to produce multiple splice isoforms (transcripts) from which the translated proteins may show differences in their expression and function [35–38]. Our study observed that at least 49% of Z-linked genes had more than two different transcripts. Unique transcript variants from multiple-transcript genes differ in male and female tissues. Moreover, our nanopore results also showed that AS is regulated in a sex and tissue manner, such that it is higher in males than females in both tissues. Therefore, we suspected that the majority of sex-specific splicing may be accounted for by tissue-specific splicing in male or female tissues, thereby implies sex-specific dosage differences of Z-linked genes in birds. In addition, Alternative polyadenylation (APA), one of the AS events, contributes to transcriptome diversity and gene expression regulation by affecting mRNA localization, stability, and translation in cells [39]. The APA has a higher frequency than splicing events. Bernardes et al. reported that the APA events are involved in the regulation of tissue-specific development [40]. Our results supported that alternative polyadenylation exhibited differences in each tissue. In summary, we believed that AS and APA achieve tissue-specific gene expression at the posttranscriptional level, with a positive regulatory role for gene dosage effects on the duck Z chromosome.

Moreover, since lncRNA regulates target gene expression and has been reported to have tissue expression specificity, their target genes and expression in different tissues were used as features of lncRNAs [41]. In this study, we identified 151 high-confidence lncRNAs as single-molecule transcripts on the duck Z chromosome. Of these, we found 28 male-biased expression lncRNAs showed a significant positive correlation with their Z-linked target gene modules and exhibiting tissue-specific expression. Studies also have reported that the lncRNA Xist (X inactive-specific transcript) has been demonstrated to regulate the dose compensation effect and X chromosome inactivation [42, 43]. Thus, we considered that the lncRNA plays a role in dosage compensation of the duck Z chromosome by regulating Z chromosome gene expression.

Notably, gene expression patterns under different conditions can often give an indication of gene function [44]. We identified the gene of male-biased, tissue-specific transcripts, and the target genes of male-biased lncRNAs were mainly enriched to cellular components and cellular response to hormone stimulus in both gonad and head skin tissues. They were mainly involved in metabolic pathways. These results indicated that the functions of the above Z-linked genes different in different cellular and physiological contexts between females and males. Interestingly, we also made novel findings with male-biased transcripts located in a 31–36 Mb region was involved biosynthesis of melanin. Melanin synthesis pathways are attributed to the high expression of multiple transcripts of a TYRP1 gene. TYRP1, one of the pigment genes, is known to be located on the Z chromosome. This gene acts as a critical downstream gene of the melanin synthesis pathway, and male ducks had significantly higher TYRP1 expression than female ducks. Therefore, we figured this gene may be one factor for the sexual dimorphism in duck feather color. From the Darwin’s theory of sexual selection, which he thought females often prefer males with brighter feather coloration, and led to a mating advantage [45, 46]. Similarly, studies by Chen x et al. indicate sexually dimorphic expression of trkB, a Z-linked gene, in early posthatch zebra finch brain; this gene plays a particularly important role in the sexual differentiation of song systems [47]. Together, these results suggested that the dosage effects of Z-linked genes related to gene function contribute to sex differences.

## Conclusions

In conclusion, our present study further explained the potential impact of posttranscriptional regulation of gene dosage effects on the avian sex chromosome. This study revealed that a dosage effect and local regulation of ducks’ Z chromosome gene expression. Additionally, our study highlights the AS and APA to achieve tissue-specific gene expression at the posttranscriptional level, with a positive regulatory role for gene dosage effects on the duck Z chromosome. The study also contributes new information regarding the role of lncRNA in the duck Z chromosome’s dosage compensation via the regulation of Z chromosome gene expression. In addition, GO enrichment and KEGG pathway analysis showed that the differences in the function of Z-linked genes contribute to sex differences.

## Materials and methods

### Ethics statement

The Animal Ethics Monitoring Committee of Sichuan Agriculture University approved all the experiments and protocols, and all methods strictly obeyed the Guide for the Animal Research: Reporting of In Vivo Experiments (ARRIVE) guidelines 2.0 [48].

### Animal materials

A total of 8 ducks (4 male and 4 female) used in the experiment were provided by the waterfowl breeding farm of Sichuan Agricultural University. By phenotypic observation, at the age of 16 weeks, the head feather color of male duck and female duck is obviously different, and a dark green head in males. Thus, animals were killed by rapid decapitation**,** we collected gonads (testis or ovary) and head skin tissues of male and female ducks, respectively. A total of 16 samples (4 for each tissue) were collected for further analysis. Of these, the head skin tissue with feather follicles around the eyes was carefully peeled off with a scalpel, and all samples were collected at the same location. After cleaning these skin tissues in PBS (phosphate buffer saline) with 0.1% DEPC (diethylpyrocarbonate) water, they were immediately stored at − 80 °C until RNA was extracted.

### RNA extraction, library construction, and sequencing

Total RNA from 4 female gonad, 4 male gonad, 4 head skin of female, and 4 head skin of male samples was respectively extracted. Total RNA with RNA integrity (RIN) number above 7.9 were considered for further analysis (Additional file 3: Table S3). See Table S1 for additional details. One microgram of total RNA was prepared for cDNA libraries using the cDNA-PCR Sequencing Kit (SQK-PCS109) protocol provided by Oxford Nanopore Technologies (ONT). Briefly, template switching activity of reverse transcriptase enriches full-length cDNAs and adds defined PCR adapters directly to both ends of the first-strand cDNA. After cDNA PCR for 14 cycles with LongAmp Tag (NEB), the PCR products were then subjected to ONT adaptor ligation using T4 DNA ligase (NEB). Agencourt XP beads were used for DNA purification according to the ONT protocol. The final cDNA libraries were added to FLO-MIN109 flow cells and run on the PromethION platform at Biomarker Technology Company (Beijing, China).

### Oxford Nanopore technologies long read processing

As output, raw reads were first filtered with a minimum average read quality score = 6 and minimum read length = 200 bp. Ribosomal RNA was discarded after mapping to the rRNA database to obtain clean reads. Next, full-length nonchemiric (FLNC) transcripts were determined by searching for primers at both ends of the reads. Clusters of FLNC transcripts were obtained after mapping to the reference genome (GCF_015476345.1, 2020) with mimimap2 (v2.16), and consensus isoforms were obtained after polishing within each cluster by pinfish. Consensus sequences were mapped to the reference genome (GCF_015476345.1, 2020) using minimap2. Mapped reads were further collapsed by cDNA_Cupcake (v5.80) with min-coverage = 85% and min-identity = 90%. A 5′ difference was not seen when collapsing redundant transcripts.

### Quantification of gene/transcript expression levels

Full-length reads were mapped to the reference transcriptome sequence. Reads with match quality above 5 were further used for quantification. Expression levels were estimated by reads per gene/transcript per 10,000 reads mapped.

### Alternative Splicing (AS) and long noncoding RNA (lncRNA) analysis

In the above obtained nonredundant transcripts, AS event identification was performed using the AStalavista tool (v3.2) [30]. The differentially spliced events were defined with at least 10% change in exon inclusion level and false discovery rate (FDR) of less than 0.05. Moreover, four approaches, including the Coding-Non-Coding-Index (CNCI, v2), Coding Potential Calculator (CPC, v1), Coding Potential Assessment Tool (CPAT, v1.2.2), and Pfam (v1.3), were utilized to sort nonprotein coding RNA candidates from the transcript’s putative protein-coding RNAs. Transcripts more than 200 nt in length and more than two exons were selected as lncRNA candidates. Relationships between lncRNAs and target genes were predicted based on their position (< 100 kb upstream or downstream) and base complementarity using the lncTar target gene prediction tool (v1.0) with default parameters [49].

### Gene functional enrichment analysis

The DAVID database was used to conduct GO enrichment analysis [50]. KOBAS [51] software was used to test the statistical enrichment of differentially expressed genes in KEGG pathways. *P* value < 0.05 was considered as significant. R language (v1.42.0) and related packages were used to visualize the results.

## Supplementary Information

**Additional file 1: Table S1.** Transcriptome full-length sequences statistics.

**Additional file 2: Table S2.** Male-biased expression of lncRNAs corresponding Z-linked target genes in gonad and in head skin tissues.

**Additional file 3: Table S3.** Sample quality check list.

**Additional file 4: Figure S1.** Rarefaction analysis of transcripts with different lengths per sample.

**Additional file 5: Figure S2.** GO and KEGG enrichment of male-biased genes for gonad (a), head skin (b). The value of log_2_ M: F (male: female) > 0. *P* < 0.05 was significant. GO enrichment on the left and KEGG enrichment on the right.

**Additional file 6: Figure S3.** Comparison transcript expression levels of autosomal and Z chromosome (CPM > 0). The M: F (male: female) ratio distributions on the Z chromosome and the autosomes for the gonad (a), head skin (b). Violin plots of the Z chromosome and the autosomes transcript expression for each tissue in males (ZZ: AA) and females (Z: AA) (c). Reads counts were converted to counts per million (CPM) values to measure the expression of transcripts. Values are represented on log_2_-transformed CPM scale (the outliers of |log_2_ FC| > 5). For simplicity, chromosome W, 31, and 33 are not included. The red lines indicate equal expression levels between females and males on the chromosome.

**Additional file 7: Figure S4.** Comparison of different transcripts among different tissues. Distribution of the number of transcripts per gene (a). Colored bars represent the number of transcripts per gene. Overlap of Z-linked transcripts in the gonad (b), head skin (c). GO and KEGG enrichment of tissue-specific transcripts for both tissues (d). *P* < 0.05 was significant. GO enrichment on the left and KEGG enrichment on the right.

**Additional file 8: Figure S5.** GO and KEGG enrichment of genes in 31–36 Mb region. GO enrichment on the left and KEGG enrichment on the right. *P* < 0.05 was significant. GO enrichment on the left and KEGG enrichment on the right.

**Additional file 9: Figure S6.** Characters of identified lncRNAs and its Z-linked target gene. Male-biased lncRNAs and their target genes cluster were identified based on their expression patterns (a). GO and KEGG enrichment of male-biased lncRNAs corresponding to Z-linked target genes for both tissues (b). *P* < 0.05 was significant. GO enrichment on the left and KEGG enrichment on the right.

**Additional file 10: Figure S7.** The M: F (male: female) ratio distributions on the Z chromosome and the autosomes of lncRNAs for the gonad (a), head skin tissues (b).

## Data Availability

The Nanopore sequencing raw data was previously released in the GSA database (https://bigd.big.ac.cn/gsa/browse/CRA004787; Accession: CRA004787).

## Abbreviations

lncRNA: Long noncoding RNAs
AS: Alterable splicing
APA: Alternative Polyadenylation
GO: Gene Ontology
KEGG: Kyoto Encyclopedia of Genes and Genomes
ONT: Oxford Nanopore Technologies
FLNC: Full-length, nonchemiric
