# Supplementary material for: Single-molecule long-read sequencing reveals the potential impact of posttranscriptional regulation on gene dosage effects on the avian Z chromosome
Source: BMC Genomics. 2022 Feb 11;23:122. doi: 10.1186/s12864-022-08360-8 (PMC8832729; doi:10.1186/s12864-022-08360-8)
Supplement: Supplementary file 1 — Additional file 1: Table S1. Transcriptome full-length sequences statistics. [file 12864_2022_8360_MOESM1_ESM.docx]

Table S1. Transcriptome full-length sequences statistics.

| **Sample ID** | **Number of clean reads (except rRNA)** | | **Number of full-length reads** | **Full-Length Percentage (FL%)** |
| --- | --- | --- | --- | --- |
| Female Gonad_1 | 3,408,338 | 2,832,189 | | 83.10% |
| Female Gonad_2 | 3,509,100 | 2,928,909 | | 83.47% |
| Female Gonad_3 | 3,833,067 | 3,207,300 | | 83.67% |
| Female Gonad_4 | 3,790,118 | 3,117,875 | | 82.26% |
| Female Head Skin_1 | 3,488,413 | 2,939,187 | | 84.26% |
| Female Head Skin_2 | 3,912,145 | 3,312,882 | | 84.68% |
| Female Head Skin_3 | 2,655,847 | 2,135,872 | | 80.42% |
| Female Head Skin_4 | 3,058,829 | 2,480,026 | | 81.08% |
| Male Gonad_1 | 2,963,878 | 2,375,658 | | 80.15% |
| Male Gonad_2 | 3,769,214 | 3,045,790 | | 80.81% |
| Male Gonad_3 | 3,340,771 | 2,660,626 | | 79.64% |
| Male Gonad_4 | 3,328,729 | 2,639,735 | | 79.30% |
| Male Head Skin_1 | 3,395,318 | 2,846,086 | | 83.82% |
| Male Head Skin_2 | 4,013,440 | 3,409,485 | | 84.95% |
| Male Head Skin_3 | 3,219,219 | 2,671,261 | | 82.98% |
| Male Head Skin_4 | 3,197,215 | 2,592,818 | | 81.10% |

Notes: The number of samples represents the biological replicates of the tissue.
