# Supplementary material for: Single-molecule long-read sequencing reveals the potential impact of posttranscriptional regulation on gene dosage effects on the avian Z chromosome
Source: BMC Genomics. 2022 Feb 11;23:122. doi: 10.1186/s12864-022-08360-8 (PMC8832729; doi:10.1186/s12864-022-08360-8)
Supplement: Supplementary file 2 — Additional file 2: Table S2. Male-biased expression of lncRNAs corresponding Z-linked target genes in gonad and in head skin tissues. [file 12864_2022_8360_MOESM2_ESM.docx]

Table S2. Male-biased expression of lncRNAs corresponding Z-linked target genes in gonad and in head skin tissues.

| **lncRNA_ID** | **Z linked Target Gene_ID** |
| --- | --- |
| ONT.23373.1 | - |
| ONT.23392.2 | AGGF1 |
| ONT.23718.1 | - |
| ONT.24208.5 | DNAJC25; GNG10; SHOC1; UGCG |
| ONT.23212.1 | ACAA2; BNC2; CZH18orf32; LIPG; MAST4; RPL17; SLC46A2; SMAD7; |
| ONT.24077.2 | KLF4 |
| ONT.23966.1 | - |
| ONT.23510.1 | NXNL2 |
| ONT.23361.2 | HTR1A |
| ONT.23667.1 | TMEM215; TOPORS |
| ONT.24045.1 | FANCC; PTCH1 |
| ONT.24170.2 | PDZD2 |
| ONT.23957.3 | MAP1B |
| ONT.24208.4 | DNAJC25; GNG10; SHOC1; UGCG |
| ONT.23392.1 | AGGF1 |
| ONT.24194.1 | - |
| ONT.23968.1 | KCNV2; VLDLR |
| ONT.24208.1 | DNAJC25; GNG10; SHOC1; UGCG |
| ONT.24279.1 | DCAF10; FRMPD1; GRHPR; POLR1E; SLC25A51; TRMT10B; ZBTB5 |
| ONT.23341.1 | ESM1 |
| ONT.24144.1 | ALDH7A1; CCDC112; FEM1C |
| ONT.24165.2 | MARVELD2; TMED7 |
| ONT.23497.1 | GAS1 |
| ONT.24166.1 | BDP1; TMED7 |
