# Supplementary material for: Single-molecule long-read sequencing reveals the potential impact of posttranscriptional regulation on gene dosage effects on the avian Z chromosome
Source: BMC Genomics. 2022 Feb 11;23:122. doi: 10.1186/s12864-022-08360-8 (PMC8832729; doi:10.1186/s12864-022-08360-8)
Supplement: Supplementary file 3 — Additional file 3: Table S3. Sample quality check list. [file 12864_2022_8360_MOESM3_ESM.docx]

Table S3. Sample quality check list

| **Sample** | **RIN value** | **28S/18S** | **Baseline** | **OD260/280** | **OD260/230** |
| --- | --- | --- | --- | --- | --- |
| Female Head Skin_1 | 8.50 | 1.81 | normal | 1.93 | 1.87 |
| Female Head Skin_2 | 8.60 | 1.92 | normal | 2.00 | 1.93 |
| Female Head Skin_3 | 8.40 | 1.78 | normal | 2.07 | 2.11 |
| Female Head Skin_4 | 8.60 | 1.72 | normal | 2.05 | 2.04 |
| Male Head Skin_1 | 8.20 | 1.60 | normal | 2.05 | 1.90 |
| Male Head Skin_2 | 8.40 | 1.65 | normal | 1.74 | 1.19 |
| Male Head Skin_3 | 7.90 | 1.49 | normal | 2.06 | 2.20 |
| Male Head Skin_4 | 8.50 | 1.68 | normal | 2.06 | 2.01 |
| Female Gonad_1 | 8.70 | 1.83 | normal | 2.05 | 2.24 |
| Female Gonad_2 | 8.40 | 1.77 | normal | 2.05 | 2.24 |
| Female Gonad_3 | 8.70 | 1.80 | normal | 1.95 | 2.12 |
| Female Gonad_4 | 8.60 | 1.68 | normal | 1.85 | 2.02 |
| Male Gonad_1 | 8.80 | 1.91 | normal | 2.05 | 2.21 |
| Male Gonad_2 | 8.60 | 1.76 | normal | 1.92 | 2.06 |
| Male Gonad_3 | 8.80 | 1.98 | normal | 1.79 | 1.96 |
| Male Gonad_4 | 8.70 | 1.71 | normal | 2.02 | 2.14 |

Notes: The number of samples represents the biological replicates of the tissue.
