# Supplementary figures and images for: Single-molecule long-read sequencing reveals the potential impact of posttranscriptional regulation on gene dosage effects on the avian Z chromosome
Source: BMC Genomics. 2022 Feb 11;23:122. doi: 10.1186/s12864-022-08360-8 (PMC8832729; doi:10.1186/s12864-022-08360-8)

## Female\_Gonad

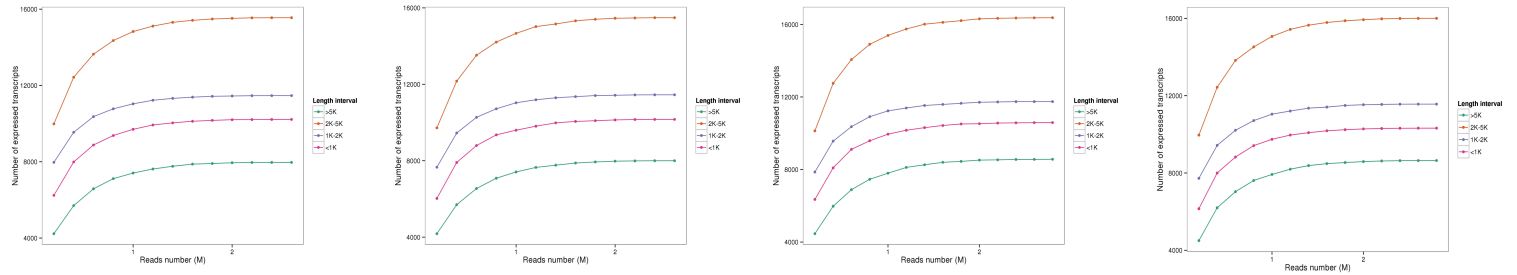

## Male\_Gonad

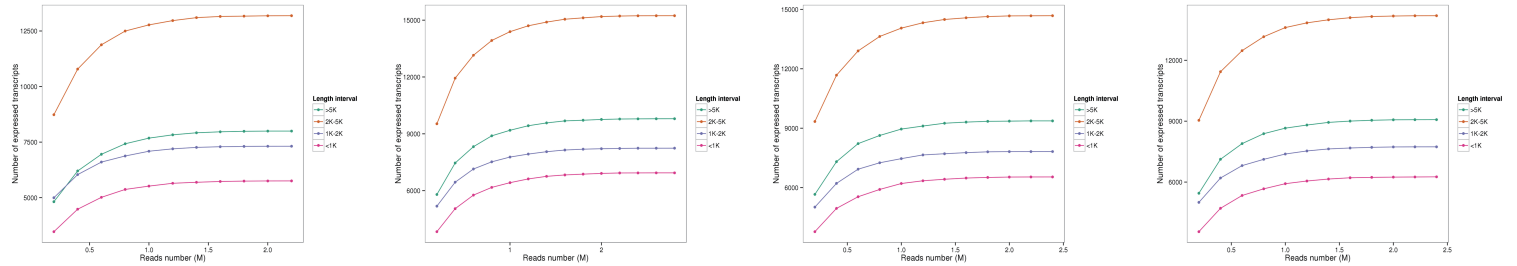

## Female\_Head skin

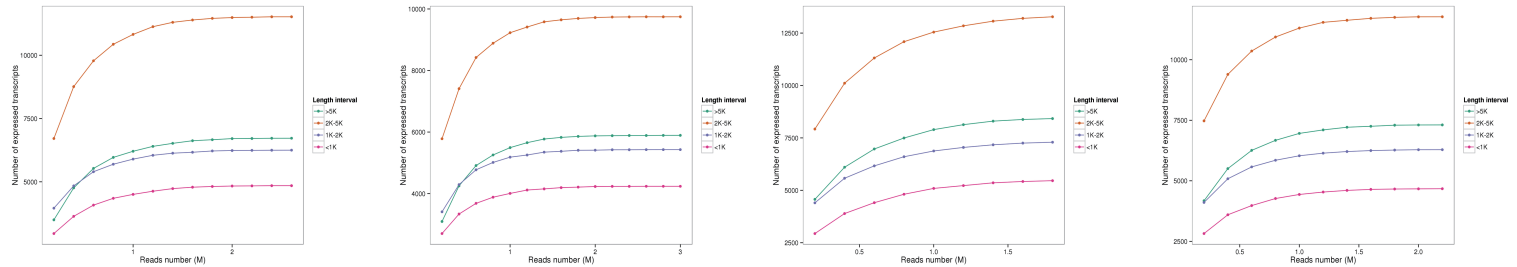

## Male\_Head skin

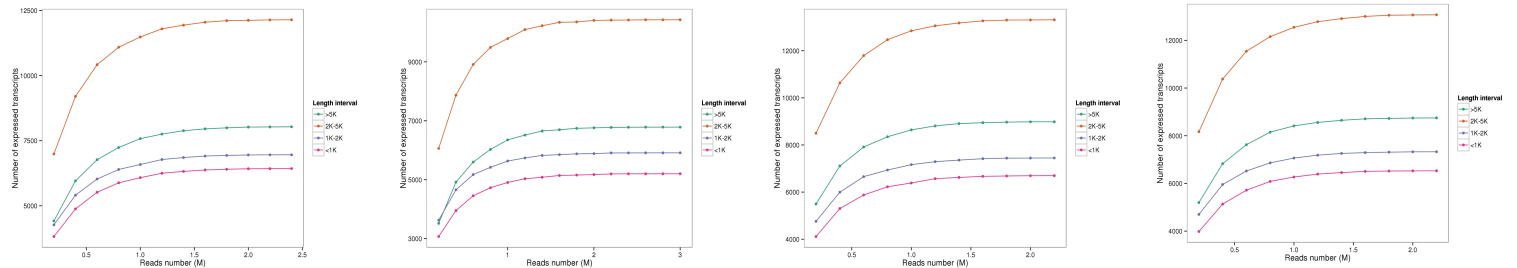

Supplement: Supplementary file 4 — Additional file 4: Figure S1. Rarefaction analysis of transcripts with different lengths per sample. [file 12864_2022_8360_MOESM4_ESM.pdf]

a

## Gonad

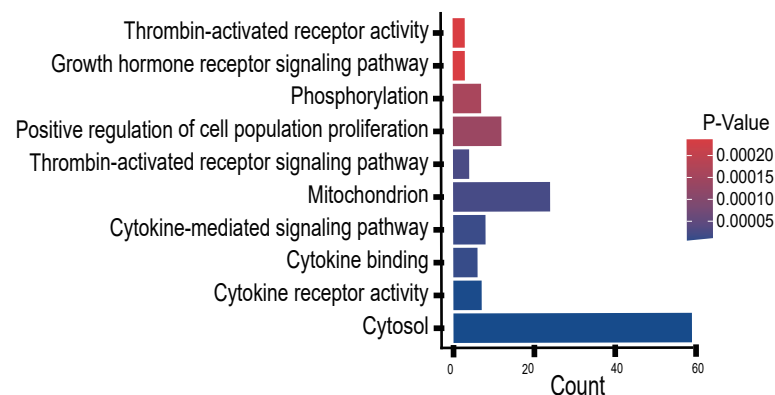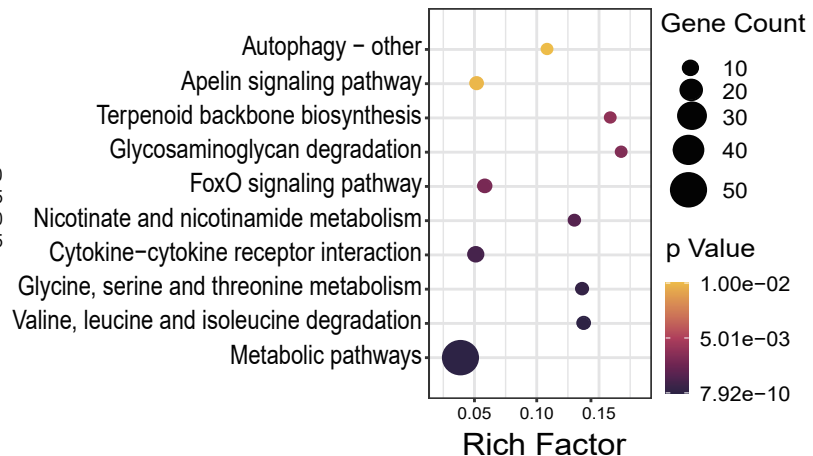

b

## Head skin

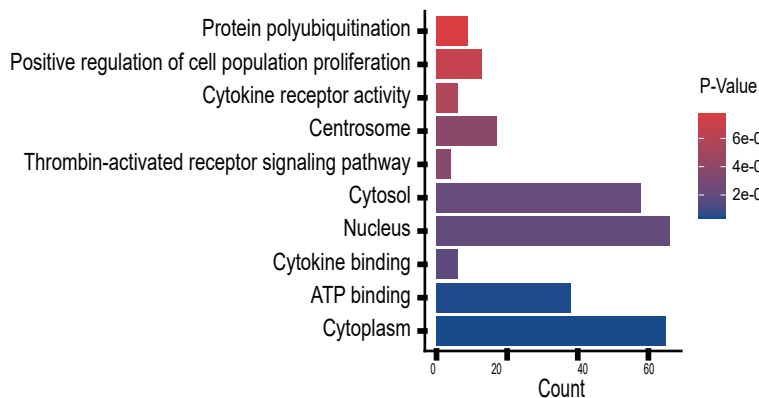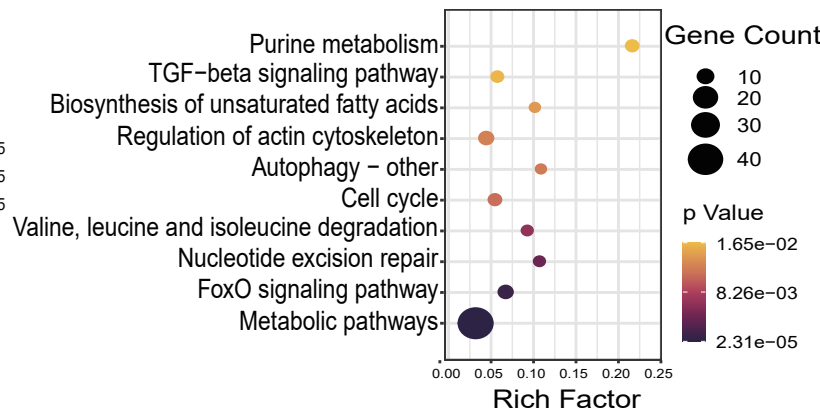

Supplement: Supplementary file 5 — Additional file 5: Figure S2. GO and KEGG enrichment of male-biased genes for gonad (a), head skin (b). The value of log2 M: F (male: female) > 0. P < 0.05 was significant. GO enrichment on the left and KEGG enrichment on the right. [file 12864_2022_8360_MOESM5_ESM.pdf]

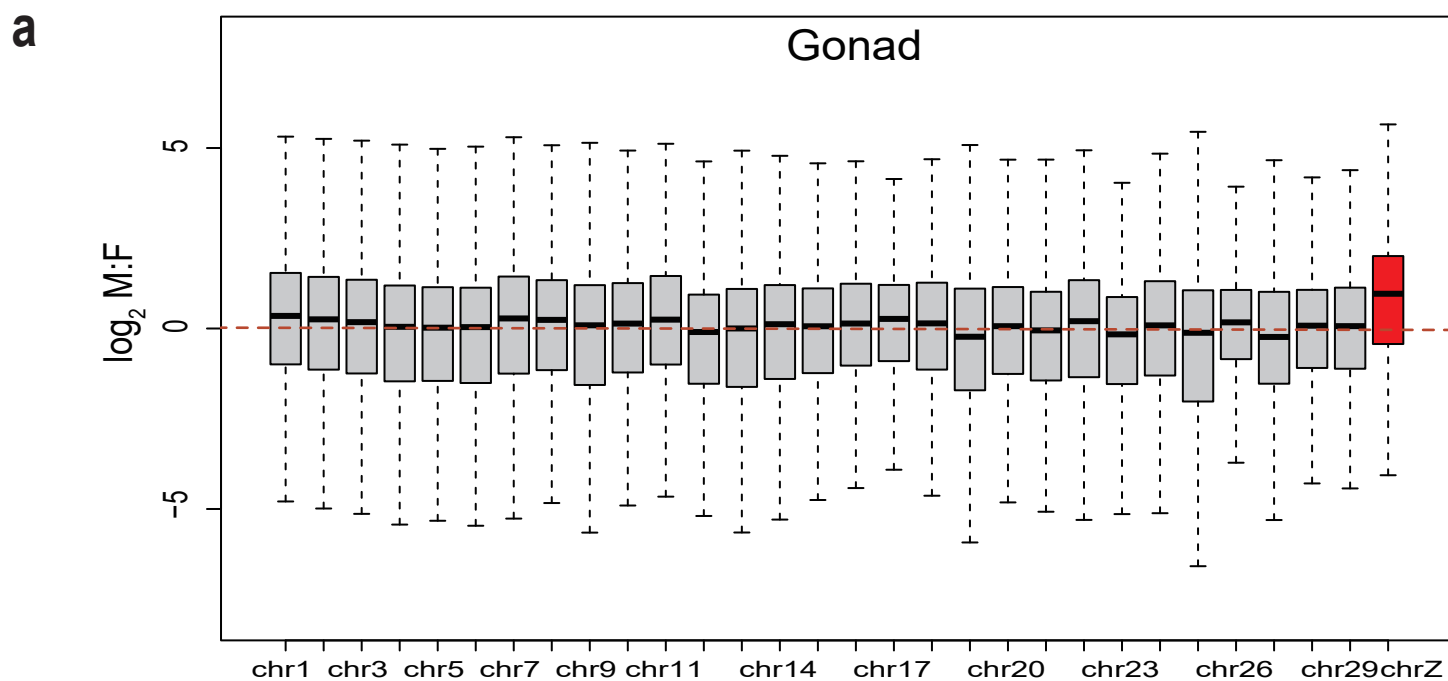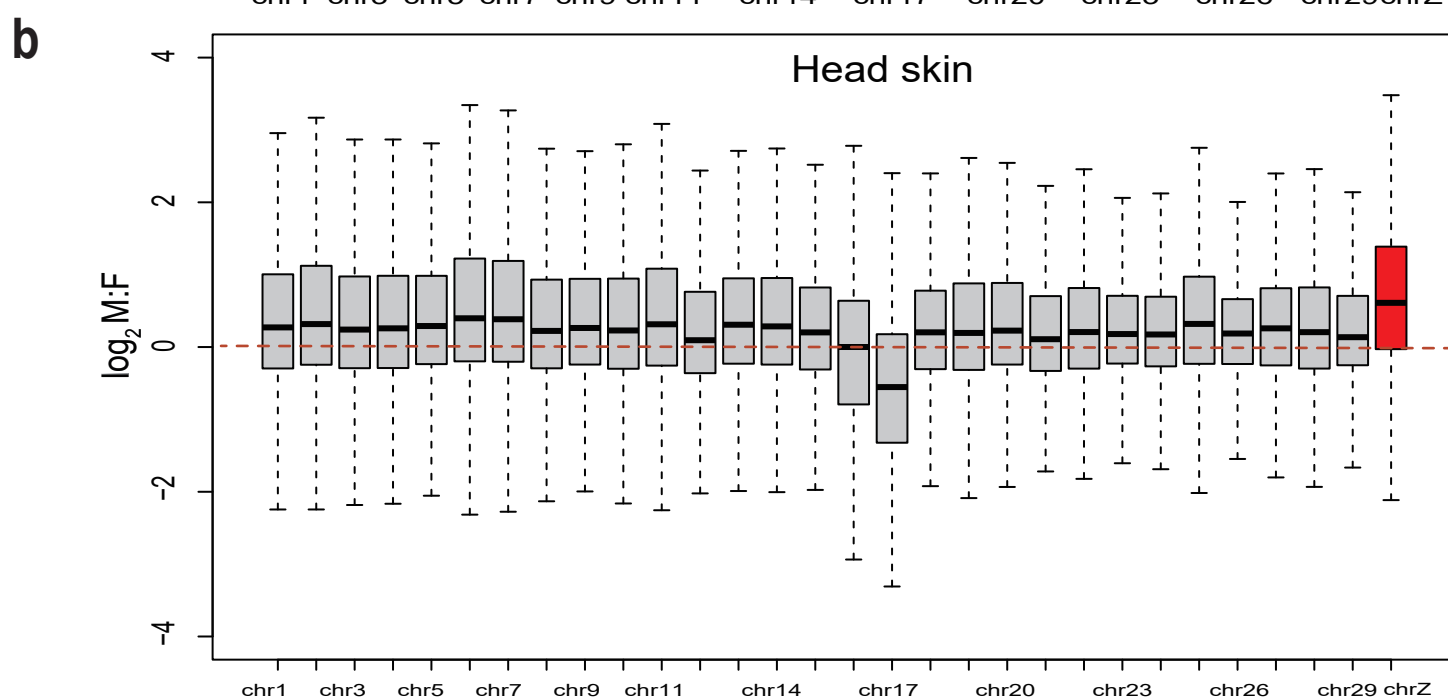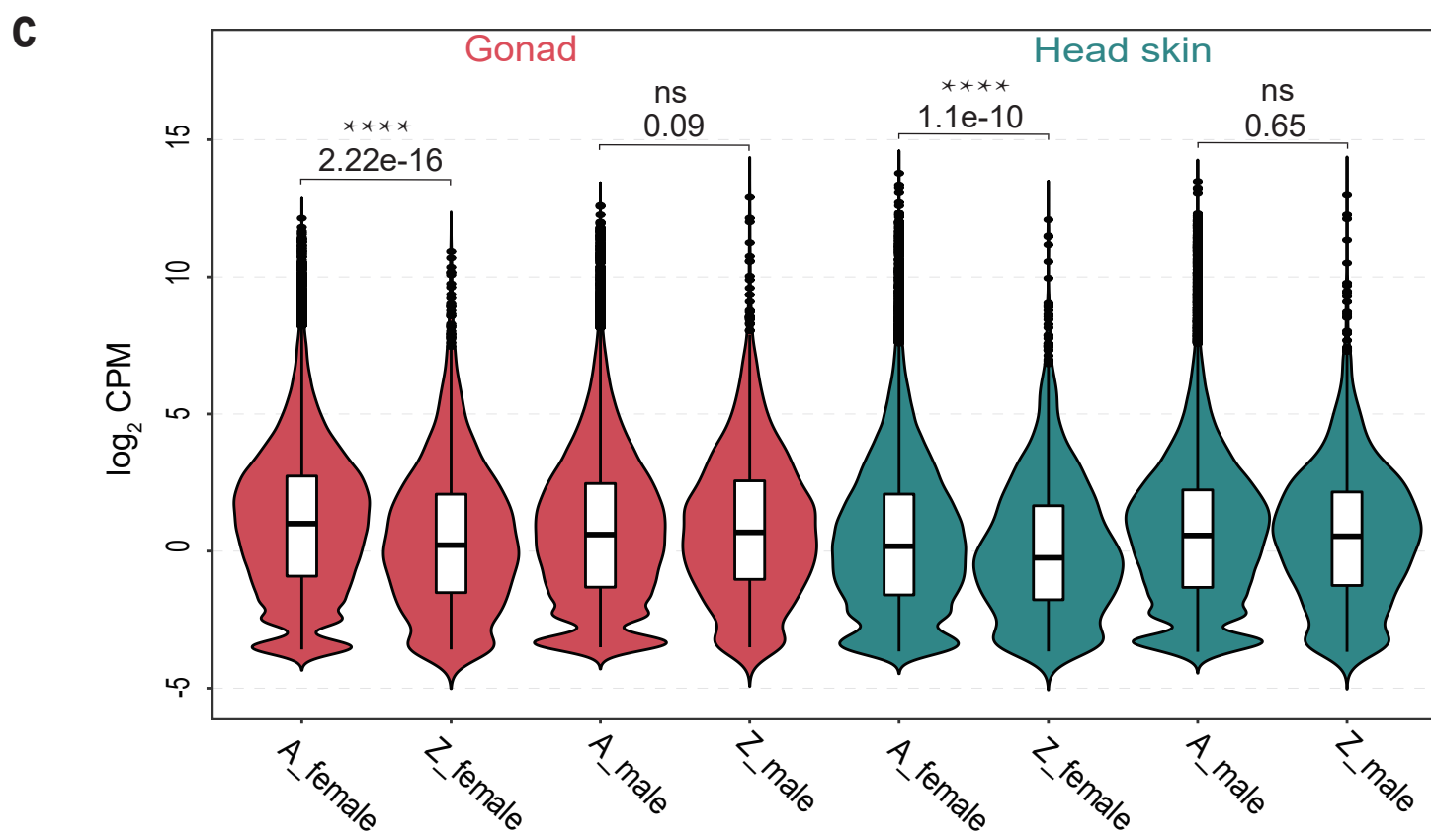

Supplement: Supplementary file 6 — Additional file 6: Figure S3. Comparison transcript expression levels of autosomal and Z chromosome (CPM > 0). The M: F (male: female) ratio distributions on the Z chromosome and the autosomes for the gonad (a), head skin (b). Violin plots of the Z chromosome and the autosomes transcript expression for each tissue in males (ZZ: AA) and females (Z: AA) (c). Reads counts were converted to counts per million (CPM) values to measure the expression of transcripts. Values are represented on log2-transformed CPM scale (the outliers of |log2 FC| > 5). For simplicity, chromosome W, 31, and 33 are not included. The red lines indicate equal expression levels between females and males on the chromosome. [file 12864_2022_8360_MOESM6_ESM.pdf]

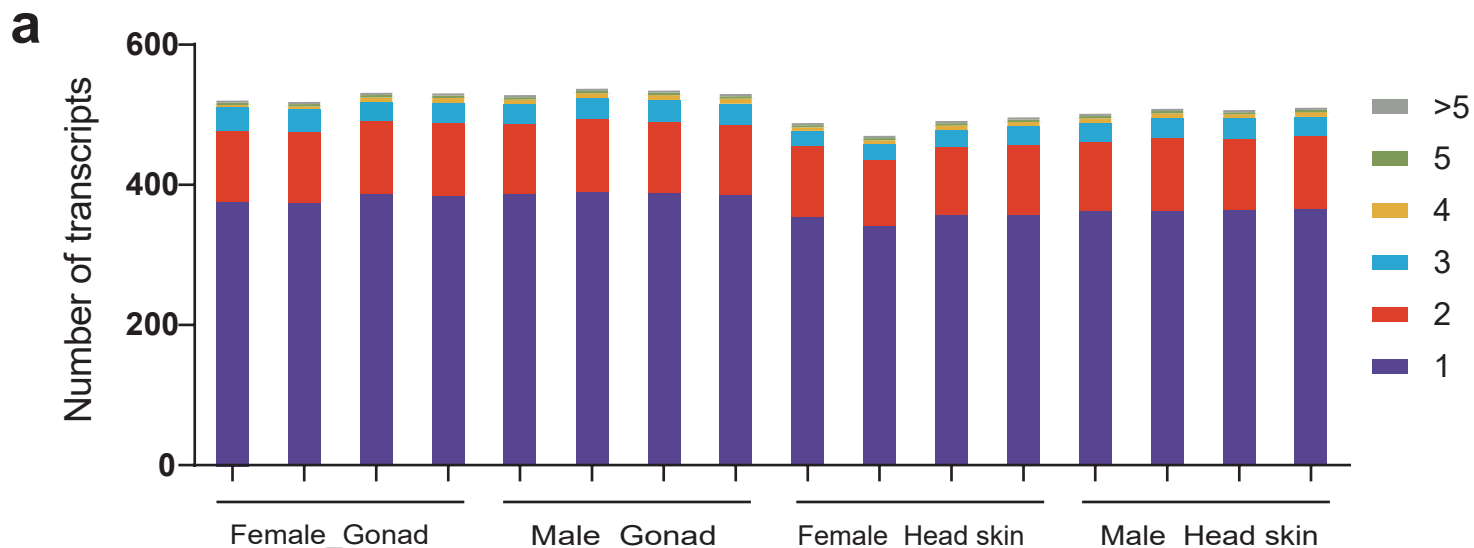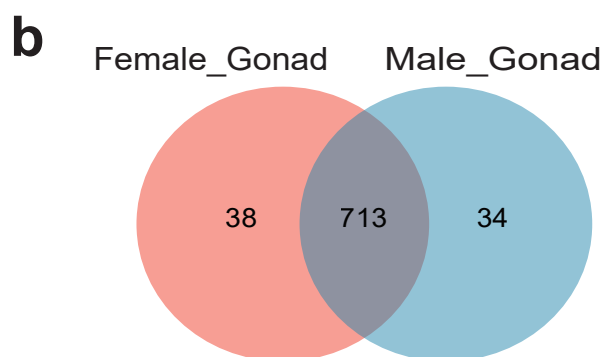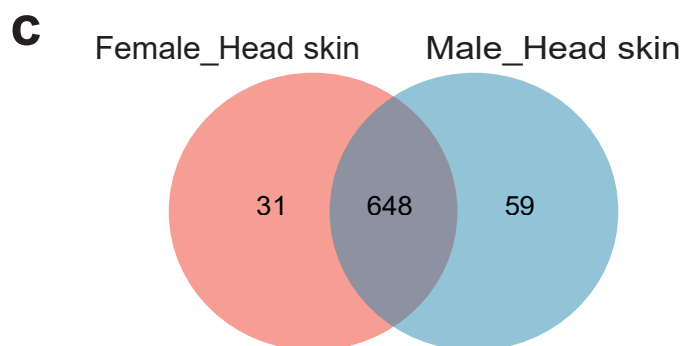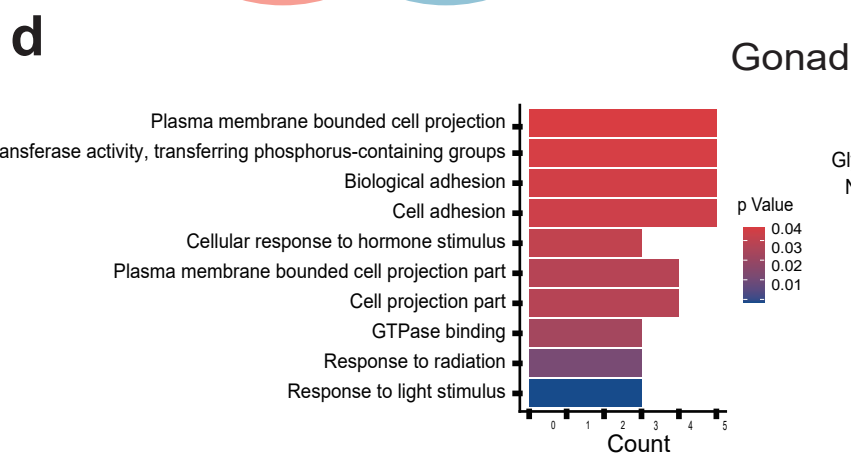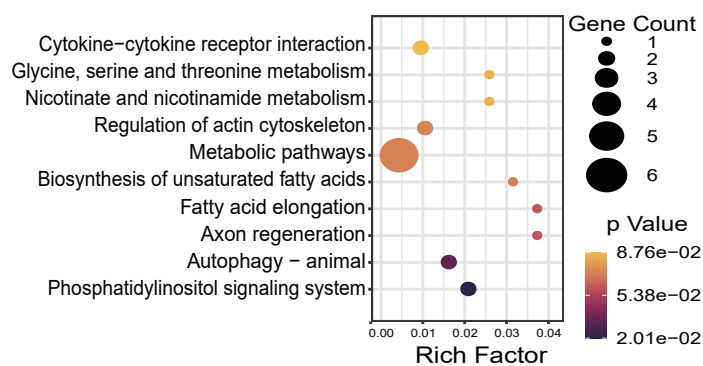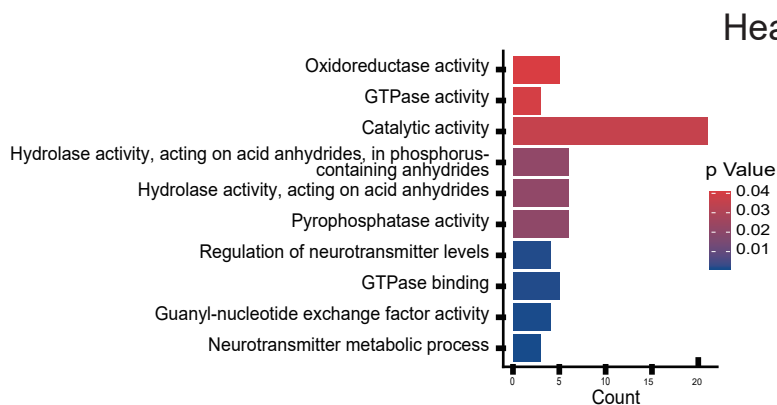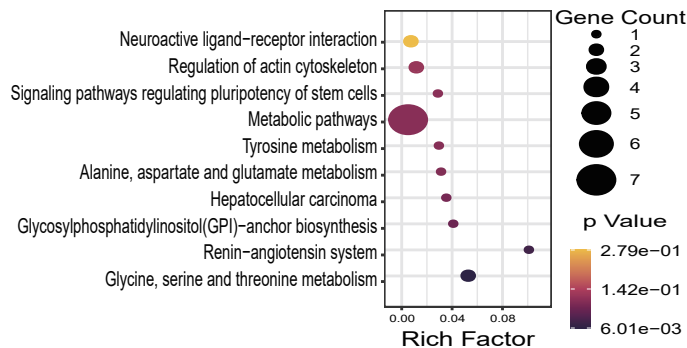

Supplement: Supplementary file 7 — Additional file 7: Figure S4. Comparison of different transcripts among different tissues. Distribution of the number of transcripts per gene (a). Colored bars represent the number of transcripts per gene. Overlap of Z-linked transcripts in the gonad (b), head skin (c). GO and KEGG enrichment of tissue-specific transcripts for both tissues (d). P < 0.05 was significant. GO enrichment on the left and KEGG enrichment on the right. [file 12864_2022_8360_MOESM7_ESM.pdf]

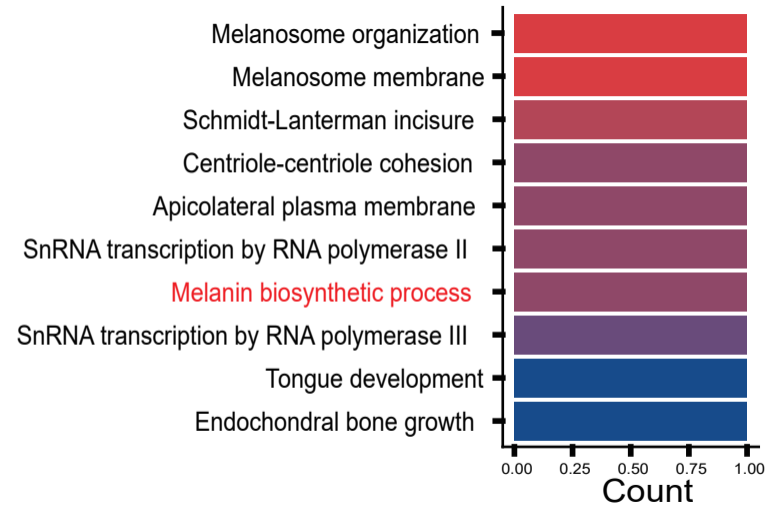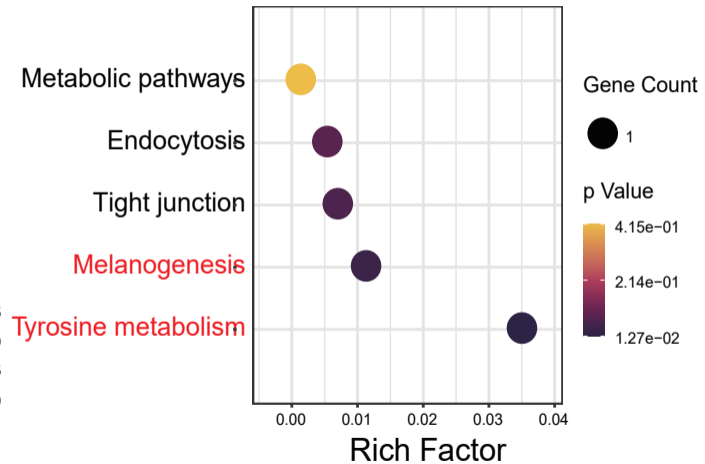

Supplement: Supplementary file 8 — Additional file 8: Figure S5. GO and KEGG enrichment of genes in 31–36 Mb region. GO enrichment on the left and KEGG enrichment on the right. P < 0.05 was significant. GO enrichment on the left and KEGG enrichment on the right. [file 12864_2022_8360_MOESM8_ESM.pdf]

**a**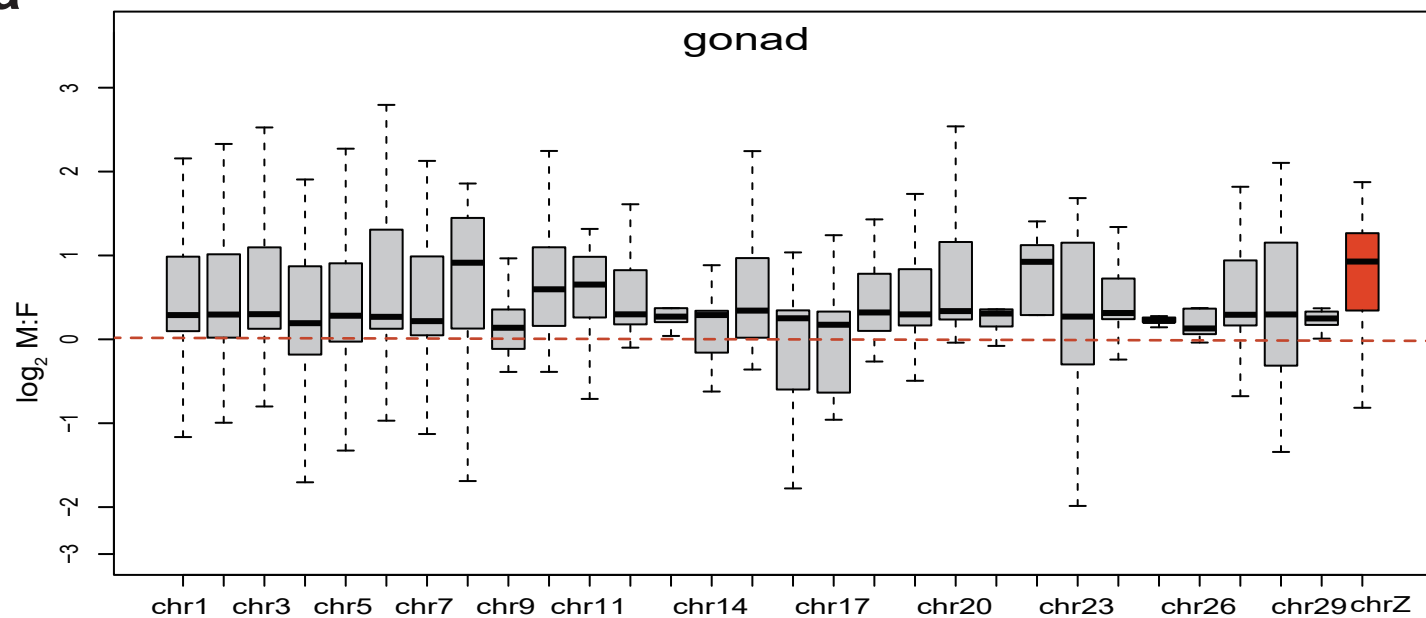**b**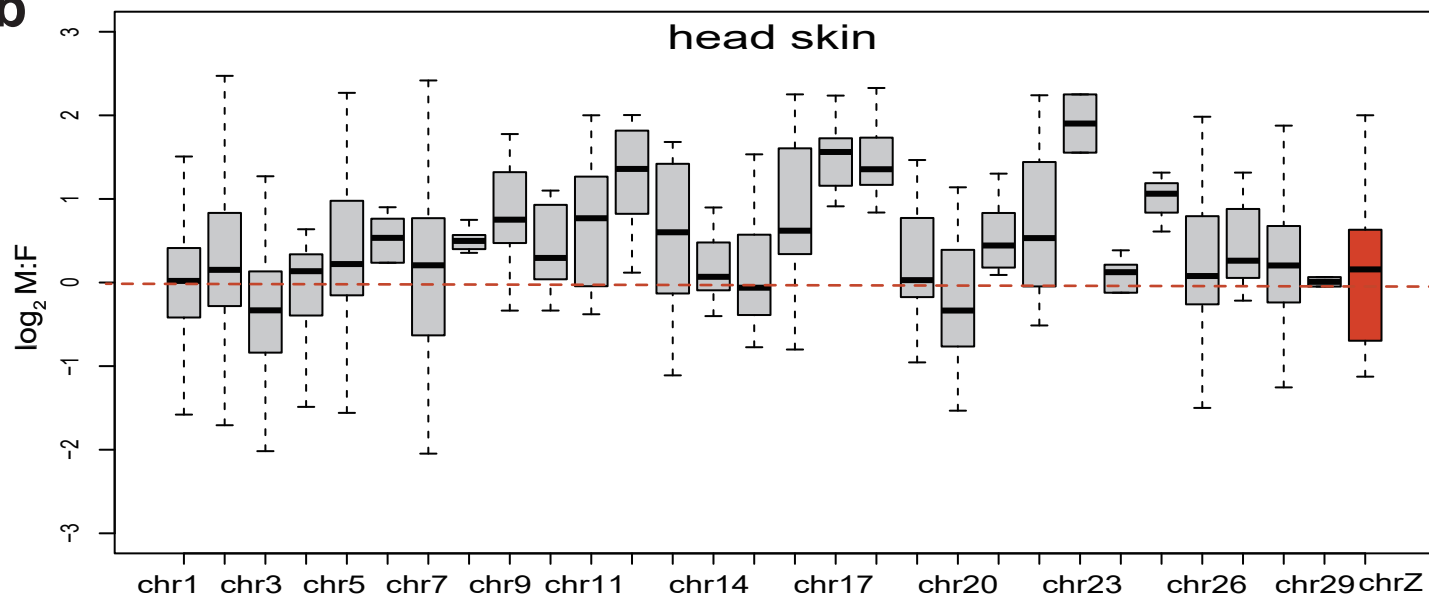

Supplement: Supplementary file 10 — Additional file 10: Figure S7. The M: F (male: female) ratio distributions on the Z chromosome and the autosomes of lncRNAs for the gonad (a), head skin tissues (b). [file 12864_2022_8360_MOESM10_ESM.pdf]
