# Supplementary material for: Single-molecule long-read sequencing reveals the potential impact of posttranscriptional regulation on gene dosage effects on the avian Z chromosome
Source: BMC Genomics. 2022 Feb 11;23:122. doi: 10.1186/s12864-022-08360-8 (PMC8832729; doi:10.1186/s12864-022-08360-8)

a

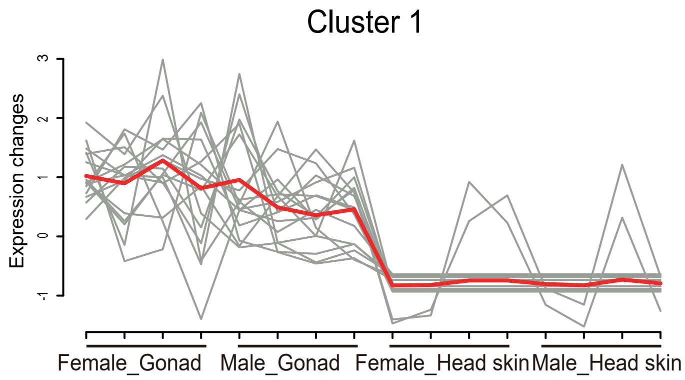

lncRNA	Target gene
ONT.23667.1	TMEM215
ONT.23968.1	VLDLR

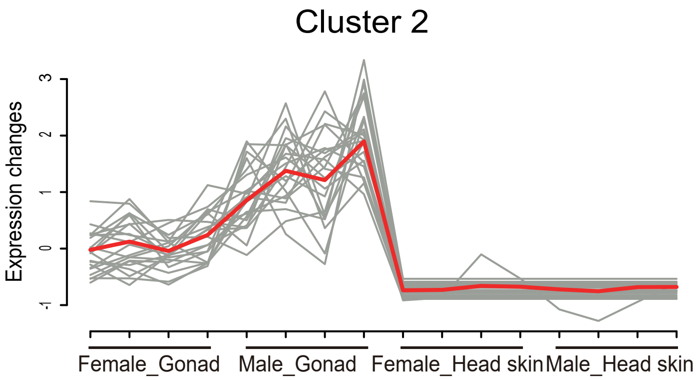

lncRNA	Target gene
ONT.24208.5	SHOC1; GNG10
ONT.23957.3	MAP1B
ONT.24208.4	GNG10; SHOC1
ONT.24208.1	GNG10; SHOC1
ONT.24279.1	DCAF10; SLC25A51
ONT.24166.1	TMED7

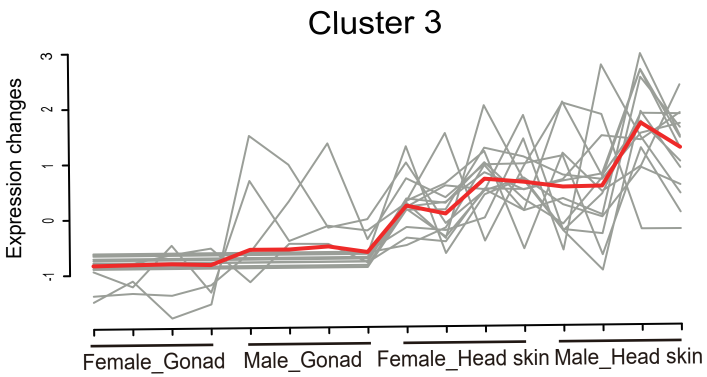

lncRNA	Target gene
ONT.24077.2	KLF4
ONT.24144.1	ALDH7A1; CCDC112; FEM1C
ONT.23497.1	GAS1

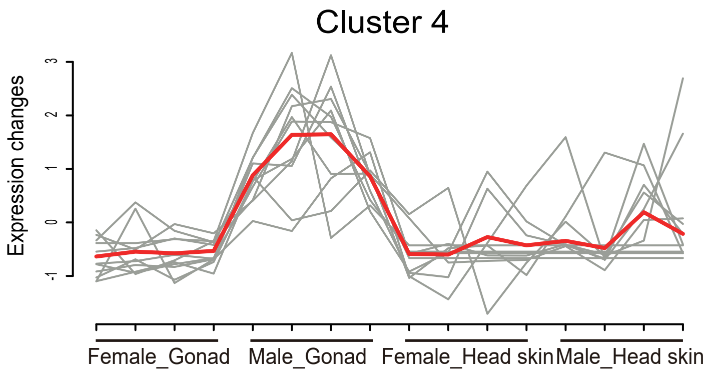

lncRNA	Target gene
ONT.23212.1	BNC2; SLC46A2
ONT.24045.1	FANCC

b

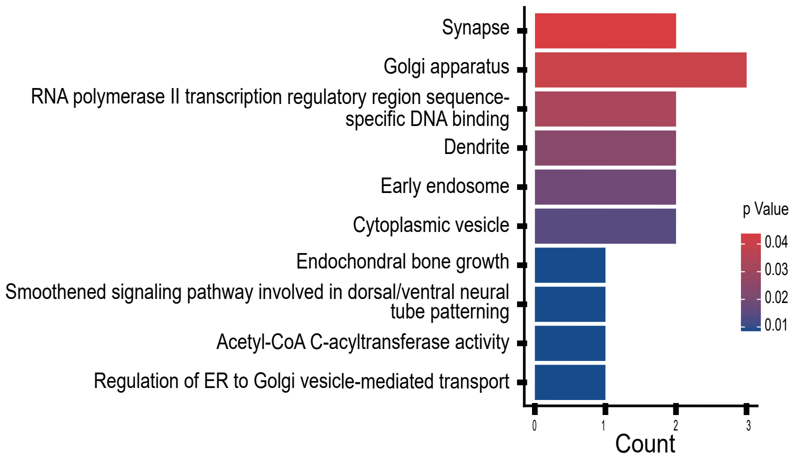

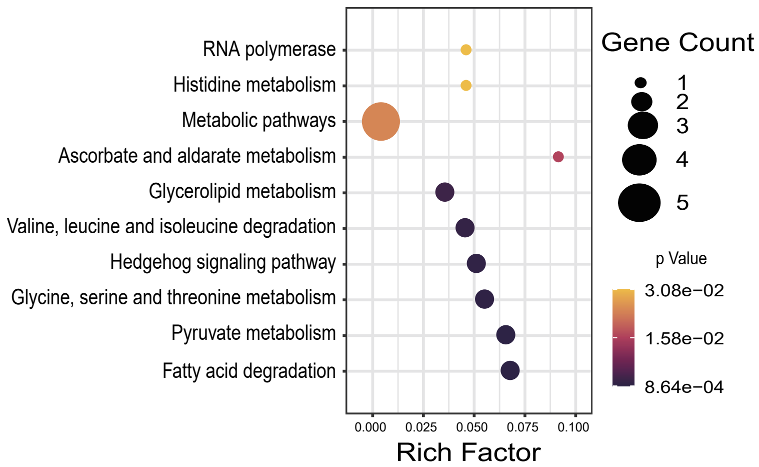

Supplement: Supplementary file 9 — Additional file 9: Figure S6. Characters of identified lncRNAs and its Z-linked target gene. Male-biased lncRNAs and their target genes cluster were identified based on their expression patterns (a). GO and KEGG enrichment of male-biased lncRNAs corresponding to Z-linked target genes for both tissues (b). P < 0.05 was significant. GO enrichment on the left and KEGG enrichment on the right. [file 12864_2022_8360_MOESM9_ESM.pdf]
